# Association of Prenatal Exposure to Maternal Drinking and Smoking With the Risk of Stillbirth

**DOI:** 10.1001/jamanetworkopen.2021.21726

**Published:** 2021-08-23

**Authors:** Hein Odendaal, Kimberly A. Dukes, Amy J. Elliott, Marian Willinger, Lisa M. Sullivan, Tara Tripp, Coen Groenewald, Michael M. Myers, William P. Fifer, Jyoti Angal, Theonia K. Boyd, Larry Burd, Jacob B. Cotton, Rebecca D. Folkerth, Gary Hankins, Robin L. Haynes, Howard J. Hoffman, Perri K. Jacobs, Julie Petersen, Nicolò Pini, Bradley B. Randall, Drucilla J. Roberts, Fay Robinson, Mary A. Sens, Peter Van Eerden, Colleen Wright, Ingrid A. Holm, Hannah C. Kinney

**Affiliations:** 1 Department of Obstetrics and Gynecology, Faculty of Medicine and Health Sciences, Stellenbosch University, Cape Town, South Africa; 2 DM-STAT Inc, Malden, Massachusetts; 3 Department of Biostatistics, Boston University School of Public Health, Boston, Massachusetts; 4 Biostatistics and Epidemiology Data Analys Center, Boston University School of Public Health, Boston, Massachusetts; 5 Center for Pediatric & Community Research, Avera Research Institute, Sioux Falls, South Dakota; 6 Department of Pediatrics, University of South Dakota School of Medicine, Sioux Falls; 7 Eunice Kennedy Shriver National Institute of Child Health and Human Development, Bethesda, Maryland; 8 Department of Psychiatry, Columbia University Medical Center, New York State Psychiatric Institute, New York; 9 Department of Pediatrics, Columbia University Medical Center, New York State Psychiatric Institute, New York; 10 Department of Pathology, Boston Children’s Hospital, Harvard School of Medicine, Boston, Massachusetts; 11 Department of Obstetrics and Gynecology, University of Texas Medical Branch, Galveston; 12 Epidemiology and Statistics Program, National Institute on Deafness and Other Communication Disorders, Bethesda, Maryland; 13 Department of Epidemiology, Boston University School of Public Health, Boston, Massachusetts; 14 Department of Pathology, University of South Dakota School of Medicine, Sioux Falls; 15 Department of Pathology, Massachusetts General Hospital, Boston; 16 PPD, Wilmington, North Carolina; 17 Department of Pathology, University of North Dakota, School of Medicine and Health Sciences, Grand Forks; 18 Department of Obstetrics and Gynecology, School of Medicine, University of North Dakota, Fargo; 19 Department of Pathology, Faculty of Medicine and Health Science, Stellenbosch University, Cape Town, South Africa; 20 Department of Pediatrics, Division of Genetics & Genomics, Manton Center for Orphan Diseases Research, Boston Children’s Hospital, Harvard Medical School, Boston, Massachusetts

## Abstract

**Importance:**

Prenatal smoking is a known modifiable risk factor for stillbirth; however, the contribution of prenatal drinking or the combination of smoking and drinking is uncertain.

**Objective:**

To examine whether prenatal exposure to alcohol and tobacco cigarettes is associated with the risk of stillbirth.

**Design, Setting, and Participants:**

The Safe Passage Study was a longitudinal, prospective cohort study with data collection conducted between August 1, 2007, and January 31, 2015. Pregnant women from Cape Town, South Africa, and the Northern Plains region of the US were recruited and followed up throughout pregnancy. Data analysis was performed from November 1, 2018, to November 20, 2020.

**Exposure:**

Maternal consumption of alcohol and tobacco cigarettes in the prenatal period.

**Main Outcomes and Measures:**

The main outcomes were stillbirth, defined as fetal death at 20 or more weeks’ gestation, and late stillbirth, defined as fetal death at 28 or more weeks' gestation. Self-reported alcohol and tobacco cigarette consumption was captured at the recruitment interview and up to 3 scheduled visits during pregnancy. Participants were followed up during pregnancy to obtain delivery outcome.

**Results:**

Of 11663 pregnancies (mean [SD] gestational age at enrollment, 18.6 [6.6] weeks) in 8506 women for whom the pregnancy outcome was known by 20 weeks’ gestation or later and who did not terminate their pregnancies, there were 145 stillbirths (12.4 per 1000 pregnancies) and 82 late stillbirths (7.1 per 1000 pregnancies). A total of 59% of pregnancies were in women from South Africa, 59% were in multiracial women, 23% were in White women, 17% were in American Indian women, and 0.9% were in women of other races. A total of 8% were older than 35 years. In 51% of pregnancies, women reported no alcohol or tobacco cigarette exposure (risk of stillbirth, 4 per 1000 pregnancies). After the first trimester, 18% drank and smoked (risk of stillbirth, 15 per 1000 births), 9% drank only (risk of stillbirth, 10 per 1000 pregnancies), and 22% smoked only (risk of stillbirth, 8 per 1000 pregnancies). Compared with the reference group (pregnancies not prenatally exposed or without any exposure after the first trimester), the adjusted relative risk of late stillbirth was 2.78 (98.3% CI, 1.12-6.67) for pregnancies prenatally exposed to drinking and smoking, 2.22 (98.3% CI, 0.78-6.18) for pregnancies prenatally exposed to drinking only after the first trimester, and 1.60 (98.3% CI, 0.64-3.98) for pregnancies prenatally exposed to smoking only after the first trimester. The adjusted relative risk for all stillbirths was 1.75 (98.3% CI, 0.96-3.18) for dual exposure, 1.26 (98.3% CI, 0.58-2.74) for drinking only, and 1.27 (98.3% CI, 0.69-2.35) for smoking only compared with the reference group.

**Conclusions and Relevance:**

These results suggest that combined drinking and smoking after the first trimester of pregnancy, compared with no exposure or quitting before the end of the first trimester, may be associated with a significantly increased risk of late stillbirth.

## Key Points

**Question:**

Is prenatal exposure to maternal drinking and smoking associated with the risk of stillbirth?

**Findings:**

In this cohort study of 8506 pregnant women (with 11 892 pregnancies) in Cape Town, South Africa, and the Northern Plains in the US, dual exposure to drinking and smoking after the first trimester of pregnancy had 2.78 times the risk of late stillbirth compared with those with no exposure or who had quit before the end of the first trimester of pregnancy.

**Meaning:**

These findings suggest that dual exposure to drinking and smoking after the first trimester of pregnancy is associated with nearly 3 times the risk of late stillbirth.

## Teaser

This cohort study assesses whether prenatal exposure to alcohol, tobacco cigarettes, or both is associated with the risk of stillbirth in pregnant women from Cape Town, South Africa, and the Northern Plains region of the US.

## Introduction

Stillbirth is a worldwide problem, with a reported overall rate in 2015 of 18.4 per 1000 births.^1^ In 2013 in the US, there were 23 596 stillbirths, with a rate of 5.96 per 1000 births.^1^ Identification of modifiable risk factors may reduce the number of these deaths.^2^ A strong association between maternal smoking during pregnancy and stillbirth has been reported.^3,4,5^ Although associations have been found for maternal drinking during pregnancy and abortion, being small for gestational age, and stillbirth,^6,7,8^ the association with stillbirth may be limited to binge drinking.^9^ Previous studies^9,10^ that assessed the association between stillbirth and maternal drinking or smoking have often been retrospective and have inadequately classified quantity, frequency, and timing of exposures. In addition, many of the stillbirth classification systems used do not sufficiently capture underlying disease processes in the exposed fetus or placenta and often use inconsistent or incomplete pathology protocols.

The Safe Passage Study, conducted by the Prenatal Alcohol in SIDS and Stillbirth (PASS) Network, is a large prospective, longitudinal cohort study conducted in populations that included women at high risk for stillbirth and sudden infant death syndrome (SIDS) and prenatal tobacco cigarette and alcohol exposure from Cape Town, South Africa, and the Northern Plains of the US.^11^ Recently, findings from the Safe Passage Study indicated that the risk of SIDS for pregnancies with combined exposure to maternal drinking and smoking were 12 times higher than the risk for pregnancies with no exposure or exposure before the end of the first trimester of pregnancy. These associations were not found in infant deaths of other known causes.^12^ The goal of the current study was to examine whether prenatal exposure to alcohol, tobacco cigarettes, or both is associated with the risk of stillbirth.^11^ Of particular interest were stillbirths of gestational age (GA) of 28 weeks or greater (late stillbirths) because this GA category is used to assess national, regional, and worldwide estimates of rates to examine trends and to determine whether the World Health Organization target of the Every Newborn Action Plan^13^ will be reached by 2030.

## Methods

The network’s steering committee and an external advisory and safety monitoring board oversaw the research. Between August 1, 2007, and January 31, 2015, a prospective cohort of 8506 women (11 892 pregnancies) were enrolled in the Safe Passage Study. In South Africa, women were recruited from 2 residential areas within Cape Town, and in the Northern Plains of the US (South and North Dakota), from 5 clinical sites, including 2 American Indian reservations. Data analysis was performed from November 1, 2018, to November 20, 2020. Ethical approval was obtained at each clinical site; Stellenbosch University, Sanford Health, the Indian Health Service, and participating Tribal Nations. Institutional review board approval, including tribal review for reservation-based sites in the Northern Plains of the US, was obtained for all PASS entities.^11,14^ Written informed consent was provided at the time of recruitment. Data were not deidentified at the time of participant recruitment but were deidentified for the purpose of data analyses. The study followed the Strengthening the Reporting of Observational Studies in Epidemiology (STROBE) reporting guideline.

### Participants

Inclusion and exclusion criteria are described elsewhere.^11^ In brief, consenting, pregnant women 16 years or older who were carrying 1 or 2 fetuses between 6 weeks’ gestation up to but not including the delivery admission and able to speak English or Afrikaans were eligible. Women planning to terminate their pregnancy or move out of the catchment area before the estimated date of delivery or were advised by a health care professional to not participate were excluded. The GA was determined during the recruitment visit using standard clinical practices: ultrasonography in South Africa and a combination of clinical examination, ultrasonography, and last menstrual period in the Northern Plains of the US. Women completed in-person study visits, which included a recruitment visit and up to 3 prenatal visits occurring at 20 to 24, 28 to 32, and 34 or more weeks of gestation, dependent on GA at enrollment. To reduce potential sources of bias, all participants presenting for prenatal care were approached for recruitment into the study, and subsequent study visits aligned with routine prenatal visits. At the recruitment visit in Cape Town, South Africa, and the Northern Plains of the US, pregnant women self-reported their racial background, choosing from the categories recommended by the National Institutes of Health: American Indian or Alaska Native, Asian, Black or African American, multiracial, Native Hawaiian or Pacific Islander, White, or other. Multiracial was defined in South Africa as having ancestry from more than 1 of the populations that inhabit the region, including Khoisan, Bantu, European, Austronesian, East Asian, or South Asian. In addition, participants self-reported their ethnic background as non-Hispanic or Latino or Hispanic or Latino. The primary goal of assessing race and ethnicity was to ensure that research can comprehensively describe the population and that findings can be generalizable.

### Outcomes

Clinical site coordinators monitored labor and delivery admissions daily to identify pregnancy outcome (ie, miscarriage, termination of pregnancy, stillbirth, and live birth). When a stillbirth occurred, the participant was asked to consent to fetal autopsy and to donate fetal brain tissue.^15^ For this analysis, the primary stillbirth outcome was defined as a fetal death delivered at 28 weeks’ gestation or later (late stillbirth ≥28 weeks), and the secondary outcome was defined as a fetal death delivered at 20 weeks or later (all stillbirth ≥20 weeks). Each stillbirth was adjudicated by a committee of multidisciplinary study investigators^16^ using the following clinical information: fetal autopsy and placental pathology (47% of cases), placental pathology only (32%), autopsy data only (5%), or clinical information only (16%). Diagnostic genetic testing was performed as indicated in 5% of cases. The most common cause of stillbirth was acute placental abruption (26%) followed by maternal vascular malperfusion (17%) and fetal vascular malperfusion (which includes umbilical cord pathology) (16%).

### Data Collection

Details of the methods used to collect and characterize alcohol and tobacco cigarette exposure were published elsewhere^17,18^ and are summarized here. Self-reported alcohol and tobacco cigarette consumption was captured at the recruitment interview and at up to 3 prenatal visits after recruitment, using a modified timeline follow-back interview for alcohol exposure and frequency and quantity of tobacco cigarettes for smoking exposure. In the event of fetal demise, exposure information was collected for the 30 days before the death. Standard drinks were calculated based on the specific alcohol content of the drinks reported. Prenatal drinking and smoking information was obtained at nearly 100% of prenatal visits. Drinking status was known for at least 6 months of the pregnancy among 94% of pregnancies and smoking status among 80% of pregnancies. Drinks per drinking-day and tobacco cigarettes per day were calculated for each month of pregnancy where exposure information was available before the stillbirth.

Group-based trajectory models were used to classify pregnancies with similar prenatal drinking patterns into 1 of 5 drinking trajectory groups (based on 11 892 pregnancies): none (48%), moderate/quit early (25%), high/quit later (10%), low continuous (12%), and high continuous (6%). Similar prenatal smoking patterns were classified into 1 of 7 smoking trajectory groups: none (52%), moderate/quit early (8%), high/quit later (2%), low continuous (10%), moderate continuous (18%), high continuous (8%), and very high continuous (2%).^18^ Because of the small number of late stillbirths (n = 82) and stillbirths (n = 145), to improve precision in the estimates of stillbirth risk associated with exposure, we dichotomized the 5-level drinking and 7-level smoking exposure measures to create a 2-level drinking and a 2-level smoking exposure measure. The 2-level drinking and 2-level smoking measures were labeled as none/quit early, defined as no exposure during pregnancy or cessation by the end of the first trimester, and continuous/quit late, defined as continuous exposure throughout pregnancy or cessation some time after the first trimester. To address the primary hypothesis that associations exist between different combinations of drinking and smoking and stillbirth risk, we created a 4-level drinking and smoking measure: none/quit early (51%), defined as no drinking or smoking during pregnancy *or* cessation by the end of the first trimester; drinking only (8%), defined as continuous/quit late for drinking *and* none/quit early for smoking; smoking only (22%), defined as continuous/quit late for smoking *and* none/quit early for drinking; and dual (19%), defined as continuous/quit late for both exposures.

### Statistical Analysis

Because stillbirth is a rare outcome, multivariable statistical approaches for adjustment were limited. Thus, propensity scores (PSs) were developed to balance the effect of nonrandom allocation of drinking and smoking exposure at baseline to increase efficiency and reduce bias caused by confounding and were included as covariates in multivariable models (K.A.D. and M.W., unpublished data, 2017). Propensity scores were developed for the 2-level and 4-level exposure measures, using baseline characteristics available on nearly the entire cohort (PS abbreviated, 2% missing) and included the following: recruitment location, maternal age, race, marital status, educational level, history of diabetes, parity, arm circumference, and statistical interactions. As a measure of nutritional status, arm circumference was used as a proxy for prepregnancy body mass index (correlation, 0.83), which was missing in 33% of pregnancies because many women did not have access to scales.^19^ An additional set of PSs were developed based on a more complete set of baseline characteristics (PS comprehensive, 19.5% missing). The PS comprehensive contained 40 baseline characteristics and statistical interactions between confounders (eTable 1 in Supplement 1).

The primary analysis set includes all pregnancies. Maternal demographic characteristics, medical and obstetric history, and infant characteristics for each stillbirth outcome, compared with live births, were expressed as risk per 1000 pregnancies. Log binomial regression using generalized linear models and generalized estimating equations to account for correlation (exchangeable) among reenrollments were used to estimate crude and adjusted relative risks to quantify associations between exposure and outcome. Adjustment in multivariable models included the PSs described above, GA at enrollment, and multifetal pregnancy. For the death outcomes, late stillbirth (≥28 weeks) and stillbirth (≥20 weeks), the primary exposure analysis included 3 planned comparisons using the 4-level drinking and smoking measure, specifically comparing the none/quit early group with the drinking only, smoking only, or dual (drinking and smoking) exposure groups, adjusted for confounding as described. Conservatively, for statistical testing that involved multiple comparisons, 2-sided 98.3% (based on Bonferroni correction) CIs were provided; otherwise, 95% CIs were provided. A 2-sided *P* < .05 was considered to be statistically significant, and 95% CIs were provided for tests of interaction. The causes of stillbirth using the 4-level drinking and smoking exposure measure were presented descriptively.

The study design and determination of sample size were described elsewhere.^11^ In brief, the study was sized for the outcome of SIDS that resulted in a sample size of 12 000 women. Assuming a sample size of 12 000, then 8 per 1000 stillbirths in the Northern Plains of the US, 15 per 1000 stillbirths in South Africa, and 49% of women prenatally exposed yields 95% power to detect a relative risk of at least 2 when comparing women with prenatal exposure with those without prenatal exposure using a χ^2^ test for proportions with continuity correction and a 2-sided *P* < .05. The study was not powered to detect effect measure modification (ie, statistical interaction between 2-level drinking and 2-level smoking main effects based on factorial design); however, the findings were provided. The study was not designed to investigate genetic and biological interactions or to perform subgroup analysis (eg, stratified by site and cause of death); however, crude assessments by site and cause of stillbirth death were provided. Analyses were performed using SAS/STAT software, version 9.4 (SAS Institute Inc).

## Results

A total of 11 892 pregnancies in 8506 women (mean [SD] gestational age at enrollment, 18.6 [6.6] weeks) were enrolled; 16% of the 8506 women enrolled more than once (range, 2-6), and 1% of the pregnancies were twins, resulting in 12 029 viable fetuses at enrollment. Delivery outcome was ascertained in 11 695 pregnancies (98%). There were 11 462 live births and 82 late stillbirths (rate, 7.1 per 1000 pregnancies) delivered 28 weeks or later and 11 518 live births and 145 stillbirths (overall rate, 12.4 per 1000 pregnancies) (Figure) delivered 20 weeks or later. A total of 37% of pregnancies were nulliparous. A total of 59% of pregnancies were in women from South Africa, 59% were in multiracial women, 23% were in White women, 17% were in American Indian women, and 0.9% were in women of other races (Table 1). Four percent reported using vasoactive substances, such as cocaine and methamphetamines, in pregnancy.

**Figure.**
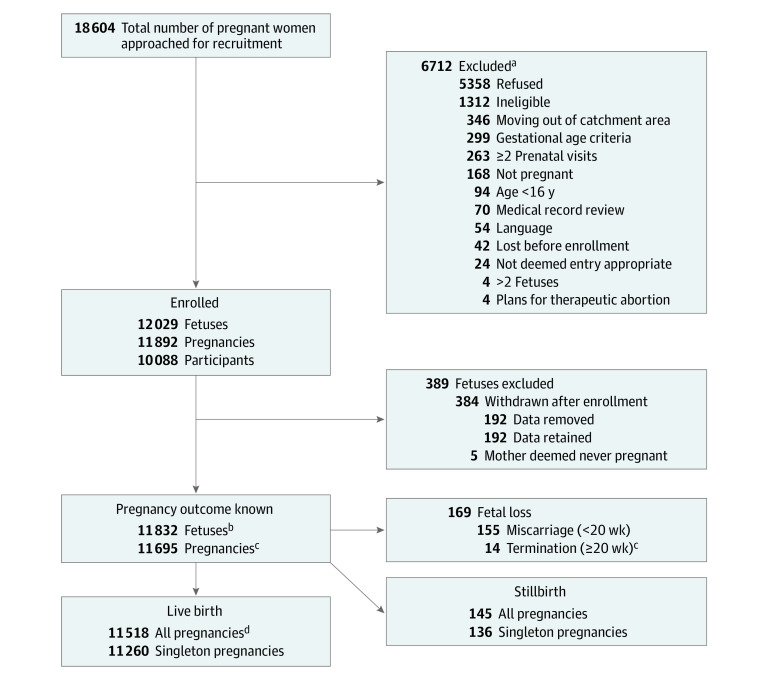
Safe Passage Study Flowchart ^a^Numbers total more than 6712 because more than 1 reason may have applied. ^b^Of those eligible for the contact. ^c^Medical indications. ^d^There were 27 congenital anomalies.

**Table 1.** Pregnancy Characteristics and Outcomes

Characteristic	Delivery at ≥20 weeks, No. (%) (n = 11 663)^a^	Delivery at ≥28 weeks			Delivery at ≥20 weeks		
		Live births, No. (n = 11 462)	Stillbirths, No. (n = 82)	Risk per 1000 pregnancies^b^	Live births, No. (n = 11 518)	Stillbirths, No. (n = 145)	Risk per 1000 pregnancies^b^
**Baseline characteristics**							
Recruitment location							
Northern Plains	4765 (41)	4706	15	3.18	4735	30	6.30
South Africa	6898 (59)	6756	67	9.82	6783	115	16.67
Maternal age, y							
<20	1902 (16)	1862	15	7.99	1873	29	15.25
20 to <35	8849 (76)	8709	57	6.50	8749	100	11.30
≥35	912 (8)	891	10	11.10	896	16	17.54
Race or ethnicity							
American Indian	1974 (17)	1943	11	5.63	1958	16	8.11
Multiracial	6871 (59)	6731	67	9.86	6758	113	16.45
White	2710 (23)	2683	4	1.49	2696	14	5.17
Other or unknown	108 (0.9)	105	0	0	106	2	18.52
Hispanic or Latino	219 (2)	213	3	13.89	215	4	18.26
Married, partnered, or living together	6803 (58)	6699	46	6.82	6727	76	11.17
Educational level							
Any primary school	646 (6)	633	8	12.48	634	12	18.58
Some high school	5506 (47)	5386	52	9.56	5418	88	15.98
Completed high school	2375 (20)	2341	16	6.79	2349	26	0.95
Beyond high school	3123 (27)	3089	6	1.94	3104	19	6.08
Prepregnancy BMI							
Underweight (BMI <18.5)	628 (8)	613	7	11.29	616	12	19.11
Normal (BMI 18.5 to <25.0)	3495 (45)	3448	17	4.91	3462	33	9.44
Overweight (BMI 25.0 to <30.0)	1832 (24)	1805	10	5.51	1815	17	9.28
Obese (BMI 30.0 to <35.0)	1007 (13)	992	7	7.01	997	10	9.93
Morbidly obese (BMI ≥35.0)	822 (11)	805	3	3.71	812	10	12.17
Arm circumference, mm							
150.0 to ≤244.5	2221 (19)	2183	15	6.82	2191	30	13.51
244.5 to ≤269.0	2343 (20)	2299	20	8.62	2309	34	14.51
269.0 to ≤294.5	2359 (20)	2313	22	9.42	2327	32	13.57
294.5 to ≤329.5	2275 (20)	2240	16	7.09	2251	24	10.55
>329.5	2287 (20)	2252	8	3.54	2263	24	10.49
Gestational age at enrollment							
First trimester (0-97 d)	2780 (24)	2724	19	6.93	2737	43	15.47
Second trimester (98-195 d)	7647 (66)	7514	53	7.00	7555	92	12.03
Third trimester (≥196 d)	1172 (10)	1162	9	7.69	1163	9	7.68
Gestational age at delivery							
<28 wk	113 (1)	0	0	0	50	63	557.52
28 wk to 31 wk 6 d	133 (1)	110	23	172.93	110	23	172.93
32 wk to 36 wk 6 d	1284 (11)	1248	36	28.04	1248	36	28.04
≥37 wk	10 127 (87)	10 104	23	2.27	10 104	23	2.27
Multifetal pregnancy	267 (2)	249	4	15.81	258	9	33.71
Medical history							
History of depression	1492 (13)	1466	9	6.10	1473	19	12.73
History of hyperthyroidism	104 (0.9)	100	1	9.90	102	2	19.23
History of hypothyroidism	277 (2)	271	2	7.33	273	4	14.44
History of diabetes	250 (2)	240	6	24.39	242	8	32.00
**Obstetric history**							
Gravidity							
1	3683 (32)	3603	31	8.53	3628	55	14.93
2	3300 (28)	3246	15	4.60	3261	39	11.82
3	2191 (19)	2163	15	6.89	2168	23	10.50
4	1267 (11)	1249	11	8.73	1252	15	11.84
≥5	1182 (10)	1163	0	0	1170	12	10.15
Parity							
0	4261 (37)	4170	35	8.32	4199	62	14.55
1	3509 (30)	3461	14	4.03	3472	37	10.54
2	2090 (18)	2061	15	7.23	2066	24	11.48
3	1040 (9)	1022	11	10.65	1027	13	12.50
≥4	723 (6)	710	7	9.76	715	8	11.07
Previous stillbirths	249 (3)	238	5	20.58	240	9	36.14
Previous miscarriages	1767 (23)	1746	9	5.13	1752	15	8.49
**Infant characteristics**							
Birth weight, g							
<1500	217 (2)	93	28	231.40	139	78	359.45
1500 to <2500	1144 (10)	1109	35	30.59	1109	35	30.59
2500 to <4000	9183 (81)	9166	17	1.85	9166	17	1.85
≥4000	826 (7)	825	0	0	826	0	0
SGA (as reported on MRA)	175 (2)	162	4	24.10	166	9	51.43
Sex							
Female	5872 (50)	5774 (50)	45 (55)	7.73	5805 (50)	67 (46)	11.41
Male	5805 (50)	5688 (50)	37 (45)	6.46	5713 (50)	78 (54)	13.47

Abbreviations: BMI, body mass index (calculated as weight in kilograms divided by height in meters squared); MRA, medical record abstraction; SGA, small for gestational age.

^a^ Of total.

^b^ (Number of Stillbirths)/(Number of Stillbirths + Number of Live Births) × 1000.

The risk of late stillbirth was 9.8 per 1000 pregnancies in South Africa, 3.2 per 1000 pregnancies in the Northern Plains of the US, and 20.6 per 1000 pregnancies in women with a prior history of stillbirth delivery (Table 1). The risk of late stillbirth in pregnancies of women less well educated was 12.48 per 1000 pregnancies in those with any primary school education, 9.56 per 1000 pregnancies in those with some high school education, 6.79 per 1000 pregnancies in those who completed high school, and 1.94 per 1000 pregnancies for those educated beyond high school. The risks of late stillbirth were 231.40 per 1000 pregnancies in women delivering infants weighing less than 1500 g, 30.59 per 1000 pregnancies for women delivering infants weighing 1500 to less than 2500 g, and 1.85 per 1000 pregnancies for women delivering infants weighing 2500 to less than 4000 g.

Given the small number of late stillbirths, we had insufficient power to rigorously assess the contribution of each unique trajectory and risk of late stillbirth; however, relative risk and CI estimates are provided (Table 2). In analysis adjusting for reenrollments, the relative risks of late stillbirth were 2.81 (95% CI, 1.54-5.11) for those pregnancies in which prenatal drinking exposure was classified as low continuous and 2.45 (95% CI, 1.19-5.03) for those classified as high/quit later compared with pregnancies identified as unexposed (5-level drinking measure) (Table 2). The relative risks of late stillbirth were 2.74 (95% CI, 1.36-5.51) for those pregnancies with smoking classified as low continuous, 2.66 (95% CI, 1.44-4.94) for those with smoking classified as moderate continuous, and 2.92 (95% CI, 1.36-6.27) for those with smoking classified as high continuous compared with pregnancies identified as unexposed (7-level smoking measure) (Table 2). The relative risks of late stillbirth were 2.51 (95% CI, 1.58-3.97) in continuous/quit late drinkers and 2.27 (95% CI, 1.41-3.66) in continuous/quit late smokers compared with those identified as none/quit early (2-level drinking and 2-level smoking measures) (Table 2). In exploratory, stratified analysis, using the 2-level exposure measures, the relative risks of late stillbirth in continuous/quit late drinkers were 1.48 (95% CI, 0.33-6.54) in the Northern Plains of the US and 2.01 (95% CI, 1.24-3.26) in South Africa compared with none/quit early drinkers. The relative risks of late stillbirth in continuous/quit late smokers were 2.54 (95% CI, 0.87-7.41) in the Northern Plains of the US and 1.58 (95% CI, 0.95-2.62) in South Africa compared with the none/quit early smokers. The relative risks of late stillbirth were 3.69 (98.3% CI, 2.05-6.67) for dually exposed, 2.53 (98.3% CI, 1.08-5.93) for drinking only, and 2.08 (98.3% CI, 1.07-4.02) for smoking only compared with those identified as none/quit early (4-level exposure measure) (Table 2).

**Table 2.** Associations Between Pregnancy Outcome and Exposure (Crude and Adjusted)

Exposure group	Pregnancies, No. (%) of total (n = 11 663)	Delivery at ≥28 weeks (n = 11 542)									Delivery at ≥20 weeks (n = 11 663)						
		Stillbirth, No. (%) (n = 82)	RR (95% CI)^a^					*P* value			Stillbirth, No. (%) (n = 145)		RR (95% CI)^a^				*P* value
**Crude associations**																	
5-Level drinking																	
None	5562 (48)	28 (0.5)	1 [Reference]					NA			63 (1)		1 [Reference]				NA
Moderate/quit early	2933 (25)	14 (0.5)	0.94 (0.49-1.80)					.85			26 (0.9)		0.73 (0.46-1.19)				.21
High/quit later	1123 (10)	15 (1)	2.45 (1.19-5.03)					.02			19 (2)		1.43 (0.81-2.52)				.22
Low continuous	1333 (11)	19 (1)	2.81 (1.54-5.11)					<.001			26 (2)		1.68 (1.04-2.73)				.04
High continuous	710 (6)	6 (0.9)	1.75 (0.73-4.21)					.21			11 (2)		1.43 (0.76-2.77)				.26
7-Level smoking																	
None	5943 (51)	25 (0.4)	1 [Reference]					NA			54 (0.9)		1 [Reference]				NA
Moderate/quit early	1030 (9)	7 (0.7)	1.67 (0.67-4.17)					.28			12 (1)		1.27 (0.65-2.49)				.48
High/quit late	394 (3)	1 (0.3)	0.55 (0.09-3.48)					.52			4 (1)		1.19 (0.44-3.17)				.74
Low continuous	1332 (11)	16 (1)	2.74 (1.36-5.51)					.005			21 (2)		1.67 (0.96-2.92)				.07
Moderate continuous	1978 (17)	22 (1)	2.66 (1.44-4.94)					.002			35 (2)		1.93 (1.23-3.02)				.004
High continuous	801 (7)	10 (1)	2.92 (1.36-6.27)					.006			15 (2)		1.97 (1.06-3.67)				.03
Very high continuous	183 (2)	1 (0.6)	1.62 (0.25-10.30)					.61			4 (2)		2.64 (1.00-6.96)				.049
2-Level drinking																	
None/quit early	8495 (73)	42 (0.5)	1 [Reference]					NA			89 (1)		1 [Reference]				NA
Continuous/quit late	3166 (27)	40 (1)	2.51 (1.58-3.97)					<.001			56 (2)		1.70 (1.19-2.43)				.004
2-Level smoking																	
None/quit early	6973 (60)	32 (0.5)	1 [Reference]					NA			66 (1)		1 [Reference]				NA
Continuous/quit late	4688 (40)	50 (1)	2.27 (1.41-3.66)					<.001			79 (2)		1.74 (1.23-2.46)				.002
2-Level drinking and 2-level smoking (in model together)^b^																	
Drinking: continuous/quit late	3166 (27)	40 (1)	2.03 (1.21-3.41)					.008			56 (2)		1.46 (1.00-2.13)				.052
Smoking: continuous/quit late	4688 (40)	50 (1)	1.80 (1.07-3.03)					.03			79 (2)		1.54 (1.06-2.23)				.23
Primary: 4-level drinking and smoking^c^																	
None/quit early	5946 (51)	22 (0.4)	1 [Reference]					NA			53 (0.9)		1 [Reference]				NA
Drinking only	1027 (9)	10 (1)	2.53 (1.08-5.93)					.03			13 (1)		1.44 (0.76-2.73)				.27
Smoking only	2549 (22)	20 (0.8)	2.08 (1.07-4.02)					.03			36 (1)		1.52 (0.96-2.41)				.07
Dual	2139 (18)	30 (1)	3.69 (2.05-6.67)					<.001			43 (2)		2.24 (1.47-3.41)				<.001
**Adjusted associations**																	
2-Level drinking and 2-level smoking (in model together)^d^^,^^e^																	
Drinking (none/quit early)	8307 (73)	41 (0.5)	1 [Reference]					NA			87 (1)		1 [Reference]				NA
Drinking (continuous/quit late)	3096 (27)	40 (1)	1.87 (1.14-3.08)					.01			56 (2)		1.31 (0.90-1.90)^e^				.16
Smoking (none/quit early)	6810 (60)	31 (0.5)	1 [Reference]								64 (0.9)		1 [Reference]				
Smoking (continuous/quit late)	4593 (40)	50 (1)	1.45 (0.78-2.67)					.24			79 (2)		1.29 (0.86-1.93)^e^				.21
Primary: 4-level drinking and smoking^c^^,^^f^																	
None/quit early	5806 (51)	21 (0.4)	1 [Reference]					NA			51 (0.9)		1 [Reference]				NA
Drinking only	1004 (9)	10 (1)	2.22 (0.78-6.18)					.06			13 (1)		1.26 (0.58-2.74)				.48
Smoking only	2501 (22)	20 (0.8)	1.60 (0.64-3.98)					.22			36 (1)		1.27 (0.69-2.35)				.35
Dual	2092 (18)	30 (2)	2.78 (1.12-6.67)					.005			43 (2)		1.75 (0.96-3.18)				.03

Abbreviations: NA, not applicable; RR, relative risk.

^a^ The 95% CIs were estimated from RRs using log binomial regression that used generalized linear models and generalized estimating equations accounting for reenrollments.

^b^ Test for interaction between 2-level drinking and 2-level smoking not significant. Estimated RR and 95% CI did not include interaction term.

^c^ The 98.3% CI estimated from RR using log binomial regression that used generalized linear models and generalized estimating equations accounting for reenrollments.

^d^ Adjusted for gestational age at enrollment, multifetal gestation, and 1 propensity score developed based on the 2-level drinking and 1 propensity score based on the 2-level smoking measures. Each propensity score included the following variables: recruitment location, maternal age, race, married or partnered status, educational level, arm circumference, history of diabetes and parity, as well as interaction terms (race × educational level, race × arm circumference, arm circumference × educational level, race × married/partnered status, and race × parity).

^e^ Propensity score based on the 2-level smoking score being removed from the model was attributable to collinearity with propensity score based on the 2-level drinking score.

^f^ Adjusted for reenrollment, gestational age at enrollment, multifetal gestation, and 3 propensity scores developed based on the 4-level exposure variable. The propensity score included the following variables: recruitment location, maternal age, race, married or partnered status, educational level, arm circumference, history of diabetes and parity, as well as interaction terms (race × educational level, race × arm circumference, arm circumference × educational level, race × married/partnered status, and race × parity).

After statistical adjustment for the abbreviated PS, GA at enrollment, multifetal pregnancy, and reenrollments, the adjusted relative risks of late stillbirth were 1.87 (95% CI, 1.14-3.08) in the continuous/quit late drinking group and 1.45 (95% CI, 0.78-2.67) in the continuous/quit late smoking group compared with those in the none/quit early group (*P* = .51 for interaction; 2-level drinking and 2-level smoking measures) (Table 2). The adjusted relative risks of late stillbirth were 2.78 (98.3% CI, 1.12-6.67) for dually exposed pregnancies, 2.22 (98.3% CI, 0.78-6.18) for drinking only, and 1.60 (98.3% CI, 0.64-3.98) for smoking only compared with those in the none/quit early group (4-level exposure measure). The adjusted relative risks of stillbirth (≥20 weeks) for pregnancies were 1.75 (98.3% CI, 0.96-3.18) for dually exposed, 1.26 (98.3% CI, 0.58-2.74) for drinking only, and 1.27 (98.3% CI, 0.69-2.35) for smoking only compared with those in the none/quit early group. The results were consistent using the comprehensive PS (eTable 2 in Supplement 1).

In 51% of pregnancies, women reported no prenatal exposure to drinking or smoking or quit before the end of the first trimester (risk of stillbirth, 4 per 1000 pregnancies). After the first trimester, 18% of mothers drank and smoked (risk of stillbirth, 15 per 1000 pregnancies), 9% drank only (risk of stillbirths, 10 per 1000 pregnancies), and 22% smoked only (risk of stillbirths, 8 per 1000 pregnancies). Dually and singly exposed pregnancies (4-level exposure measure) differed in their patterns of drinking and smoking (5-level drinking and 7-level smoking measure); those reporting dual exposure drank and smoked more than those reporting single exposure. For example, among dually exposed pregnancies, 45% were low continuous and 27% were high continuous drinkers compared with 37% low continuous and 13% high continuous drinkers in those only singly exposed to drinking; the proportion of high/quit late drinkers was approximately half that in dually exposed pregnancies compared with drinking only (29% vs 49%). Among the dually exposed, 45% were moderate continuous and 23% were high continuous or very high continuous smokers compared with 40% of moderate continuous and 19% of high continuous or very high continuous smokers in the smoking-only group.

The stillbirths analyzed in this report (n = 145) include antepartum (71%), intrapartum (15%), and stillbirth unknown (14%). A total of 10% were of undetermined cause, and 12% had insufficient information to classify. The proportion of stillbirths attributed to acute abruption was almost 2 times higher among women reporting dual (30%) or smoking-only (31%) exposure during pregnancy compared with those with none/quit early exposure (19%). The proportion of stillbirths attributed to ascending infection was at least twice that for women reporting dual (19%) or drinking-only (15%) exposure compared with women reporting none/quit early exposure (8%) during pregnancy. In addition, an undetermined cause of the stillbirth was more common among women who reported any exposure compared with none/quit early (15% drinking only, 14% smoking only, and 12% dual compared with 6%) (Table 3).

**Table 3.** Exposure by Cause of Stillbirth^a^

Cause of stillbirth	No. (%) of stillbirths				
	Total (n = 145)	4-Level exposure (% of exposure total)			
		None/quit early	Drinking only	Smoking only	Dual
Acute abruption	37 (26)	10 (19)	3 (23)	11 (31)	13 (30)
Chronic placental perfusion failure	24 (17)	9 (17)	1 (8)	6 (17)	8 (19)
Cord pathology (FVM)	23 (16)	11 (21)	3 (23)	8 (22)	1 (2)
Insufficient information	17 (12)	5 (9)	1 (8)	5 (14)	6 (14)
Undetermined	15 (10)	3 (6)	2 (15)	5 (14)	5 (12)
Ascending infection	14 (10)	4 (8)	2 (15)	0	8 (19)
Hematogenous infection	6 (4)	3 (6)	0	1 (3)	2 (5)
Intrinsically fetal	5 (3)	4 (8)	1 (8)	0	0
Other	4 (3)	4 (8)	0	0	0

Abbreviation: FVM, fetal vascular malperfusion.

^a^ Prenatal Alcohol in SIDS and Stillbirth classification.

## Discussion

In seeking modifiable behaviors for stillbirth prevention, this cohort study focused on the estimation of the associations among drinking, smoking, and dual exposure in pregnancy and the risk of stillbirth. After statistical adjustment, participants dually exposed had nearly 3 times the risk of late stillbirth compared with pregnancies not exposed or in mothers quitting after the first trimester. Considering the factorial model, with both the 2-level smoking and 2-level drinking measures included, the adjusted relative risks of late stillbirth were 1.87 (95% CI, 1.14-3.08) in women reporting continuous/quit late drinking and 1.45 (95% CI, 0.78-2.67) in women reporting continuous/quit late smoking compared with those reporting none/quit early (*P* = .51 for interaction between drinking and smoking). Similar associations were observed for stillbirth at 20 weeks or later but did not reach statistical significance. If analyses had ended with the traditional 2 × 2 factorial model for effects of drinking and smoking using the 2-level exposure measures and the interaction, the importance of dual exposure may have been missed. This new finding, to our knowledge, of an association between late stillbirth attributable to combined prenatal exposures to drinking and smoking is important given the extensive documentation that alcohol is frequently used in combination with tobacco cigarettes,^20^ including by pregnant women.

As to potential mechanisms whereby the 2 exposures lead to increased risk, combined drinking and smoking are also associated with an increased frequency of low birth weight than either substance alone. A previous study^21^ postulated that there may be an association in the growth-limiting effects of both exposures and an increase in health-compromising behaviors. This same group reported a similar effect on preterm labor and suggested the mechanism for dual exposure is still unclear.^22^ Both prenatal smoking and drinking exposures are associated with placental syndromes, pathological states arising from diseased placental spiral arteries, placental ischemia, and endothelial dysfunction.^23,24^ Concomitant smoking and drinking increased the risk of small for gestational age.^11^ These authors suggested that this combined effect is probably attributable to the negative effect of both drugs on fetal growth. The more severe effects of concomitant use, as found in our study and the study by Aliyu et al,^10^ are supported by a previous review^25^ of these dual exposures.

Several episodes of binge drinking during pregnancy are reported to increase the odds of congenital cardiac defects, and the association is more pronounced when combined with maternal smoking.^26^ Simultaneous exposure also seems to increase the odds of esophageal atresia in contrast to smoking or drinking only.^27^ Because the use of drinking or smoking during pregnancy has effects on several micronutrients, nutritional deficiency could also underlie their combined effect.^28^

### Strengths and Limitations

This study has strengths, including (1) prospective collection of prenatal drinking and smoking consumption repeated throughout pregnancy for nearly 100% of pregnancies; (2) ascertainment of pregnancy outcome for almost the total cohort (98%); (3) standardized pathological evaluation (fetal autopsy and placental examination) and classification of most of the stillbirths by a multidisciplinary committee; and (4) a large cohort of mothers at high risk, providing a sufficient sample size for statistical analysis.

This study also has several limitations. First, exposure status of the women was based on self-report, and it is possible that some women were misclassified. Because exposure was collected prospectively, the misclassification should be similar for pregnancies resulting in stillbirth or live birth, giving more confidence to our estimates of risk. In addition, the analyses reported did not account for the potential contribution of passive smoke. Second, data on quantity, frequency, and timing of illicit drug use were not collected, only date of last use. Furthermore, based on a priori study design, the Northern Plains of the US and South African cohorts were combined. In stratified analysis by site, the unadjusted risk of late stillbirth and the 2-level drinking measure in pregnancies of women who reported continuous/quit late drinking were at least 1.5 times those reporting none/quit early; the same association was found for the 2-level smoking measure. We cannot rule out the possibility of residual confounding attributable to illicit drug use, site, or other unmeasured factors. Third, stillbirth is a relatively rare outcome and presents challenges for precise estimation of effects and appropriate control for confounding. Fourth, the study cohort was selected to include populations with documented high rates of stillbirth and prenatal exposure to drinking and smoking. The generalizability of the findings must consequently be taken into consideration. However, the results remained significant after adjustment for other known risk factors for stillbirth, including socioeconomic status and educational level, suggesting relevance for other populations.

## Conclusions

Among pregnant women in Cape Town, South Africa, and the Northern Plains of the US, combined drinking and smoking after the first trimester of pregnancy compared with no exposure or quitting before the end of the first trimester were associated with the risk of late stillbirth. This new finding of the association between stillbirth and combined prenatal exposures to drinking and smoking is important given the extensive documentation that alcohol is frequently used in combination with tobacco cigarettes, including by pregnant women.

## Notes

Supplement 1. **eTable 1.** Baseline Characteristics Collected in the Safe Passage Study Hypothesized to Confound or Modify the Relationship Between Prenatal Drinking and Smoking and Perinatal Outcomes (Comprehensive Propensity Scores) **eTable 2.** Late Stillbirth and 4-Level Drinking and Smoking Exposure Measure, Adjusted for Comprehensive Propensity Score

Supplement 2. **Nonauthor Collaborators.** The Prenatal Alcohol in SIDS and Stillbirth (PASS) Network Members
