# Supplementary material for: Association of Prenatal Exposure to Maternal Drinking and Smoking With the Risk of Stillbirth
Source: JAMA Netw Open. 2021 Aug 23;4(8):e2121726. doi: 10.1001/jamanetworkopen.2021.21726 (PMC8383134; doi:10.1001/jamanetworkopen.2021.21726)
Supplement: Supplement 1. — eTable 1. Baseline Characteristics Collected in the Safe Passage Study Hypothesized to Confound or Modify the Relationship Between Prenatal Drinking and Smoking and Perinatal Outcomes (Comprehensive Propensity Scores) eTable 2. Late Stillbirth and 4-Level Drinking and Smoking Exposure Measure, Adjusted for Comprehensive Propensity Score [file jamanetwopen-e2121726-s001.pdf]

Supplementary Online Content

Odendaal H, Dukes KA, Elliott AJ, et al. Association of prenatal exposure to maternal drinking and smoking with the risk of stillbirth. *JAMA Netw Open*. 2021;4(8):e2121726. doi:10.1001/jamanetworkopen.2021.21726

eTable 1. Baseline Characteristics Collected in the Safe Passage Study Hypothesized to Confound or Modify the Relationship Between Prenatal Drinking and Smoking and Perinatal Outcomes (Comprehensive Propensity Scores)

eTable 2. Late Stillbirth and 4-Level Drinking and Smoking Exposure Measure, Adjusted for Comprehensive Propensity Score

This supplementary material has been provided by the authors to give readers additional information about their work.

© 2021 Odendaal H et al. *JAMA Network Open.*

eTable 1. Baseline Characteristics Collected in the Safe Passage Study Hypothesized to Confound or Modify the Relationship Between Prenatal Drinking and Smoking and Perinatal Outcomes (Comprehensive Propensity Scores)

**Category**	**Variables[a]**	**Method (Collection Time Point)**
Demographics	**recruitment center, age at enrollment, race, ethnicity, marital status, education**	Self-report (Recruitment)
Socioeconomics	**employment status, monthly household income, crowding index, access to: running water, toilet or electricity inside the home, working phone or cell service, number of moves in the past year, health care coverage, including subsidies and other government support**	Self-report (1st Prenatal Visit*)
Medical History	**depression, anxiety, hypertension, diabetes, hepatitis, tuberculosis, anemia, blood clotting disorder/stroke,** human immunodeficiency virus **, epilepsy, heart disease, thromboembolic disease, hyperthyroidism, hypothyroidism, renal disease, cancer and autoimmune disorders, including antiphospholipid antibody syndrome, lupus erythematosus, rheumatoid arthritis, connective tissue disorder, sickle cell anemia**	Self-report (1st Prenatal Visit) confirmed with Medical Record (abstracted after delivery)
Obstetric and Reproductive History	**gravidity, parity, fertility treatment for the index pregnancy**, and among multigravida women, obstetric characteristics, complications and outcomes of prior pregnancies, i.e., gestational diabetes, pregnancy-induced hypertension, preeclampsia, placenta praevia, placental abruption and preterm premature rupture of membranes, cesarean delivery, preterm birth, intrauterine growth restriction or small for gestational age, hypoxia/encephalopathy, jaundice, miscarriage, abortion, stillbirth, heart defects, neural tube defects, shoulder dystocia, neonatal intensive care unit admission, poor weight gain, SIDS or sudden unexplained infant death, child with diagnosed or suspected Down or fetal alcohol syndrome	Self-report (1st Prenatal Visit) confirmed with Medical Record (abstracted after delivery)
Indicators of Nutritional Status	prepregnancy weight<br>maternal height and **arm circumference**<br>body-mass-index (BMI), calculated from height and weight	Self-report (Recruitment)<br>Measured by study personnel (Recruitment)
**Exposure History**	**frequency of drinking and smoking year prior to pregnancy**	**Self-report (Recruitment)**

[a] Bolded items were used in the comprehensive propensity scores. All other items were considered for inclusion, but were not used

© 2021 Odendaal H et al. *JAMA Network Open.*

eTable 2. Late Stillbirth and 4-level Drinking and Smoking Exposure Measure, Adjusted for Comprehensive Propensity Score [a]

Exposure Group	Pregnancies n=9,294[b]	Pregnancies delivered ≥ 28 weeks n=11,542		
		Stillbirth n=55	RR (98.3% CI)[c]	p-value
None	4860 (52.3%)	16 (0.4%)	1.0	
Drinking-only	788 (8.5%)	8 (1.0%)	2.12 (0.61,7.36)	0.151
Smoking-only	2001 (21.5%)	13 (0.7%)	1.44 (0.51, 4.11)	0.403
Dual	1645 (17.7%)	18 (1.1%)	2.17 (0.72, 6.55)	0.095

[a]Adjusted for reenrollment, gestational age at enrollment, multi-fetal gestation and three propensity scores developed based on the 4-level exposure variable. The propensity score included the variables listed in eTable1
[b]Of total
[c]98.3% CI adjusted for Bonferroni correction α=0.0167, estimated from RR using log binomial regression utilizing generalized linear models and generalized estimating equations accounting for reenrollments

RR: Relative Risk, CI: Confidence Interval

Primary Exposure Measure: 4-level Drinking and Smoking Measure
-None: No smoking *or* drinking during pregnancy or quitting by the end of the 1st trimester
-Drinking-only: Drinking after the 1st trimester of pregnancy *and* no smoking or quitting by the end of the 1st trimester
-Smoking-only: Smoking after the 1st trimester of pregnancy *and* no drinking or quitting by the end of the 1st trimester
-Dual: Drinking *and* Smoking after 1st trimester of pregnancy

© 2021 Odendaal H et al. *JAMA Network Open.*
