# Supplementary material for: Association of Prenatal Exposure to Maternal Drinking and Smoking With the Risk of Stillbirth
Source: JAMA Netw Open. 2021 Aug 23;4(8):e2121726. doi: 10.1001/jamanetworkopen.2021.21726 (PMC8383134; doi:10.1001/jamanetworkopen.2021.21726)
Supplement: Supplement 2. — Nonauthor Collaborators. The Prenatal Alcohol in SIDS and Stillbirth (PASS) Network Members [file jamanetwopen-e2121726-s002.pdf]

Supplemental Online Content: Nonauthor Collaborators

*Indicates required information. Only first name, last name, and suffix will appear in PubMed.

*Group Name(s): the PASS Network

*First Name and Middle Initial(s)	*Last Name	*Suffix (eg, Jr, III)	Academic Degrees	Institution	Location (city, state/province, country)	Role or Contribution, eg, chair, principal investigator	Group (if more than 1 Group listed in the byline) and/or Subgroup (eg, Steering Committee)
Travis	Baker		BS	DM-STAT, Inc.	Malden, MA, USA		
Rebecca A.	Young		MPH	DM-STAT, Inc.	Malden, MA, USA		
Idania	Ramirez		MPH	DM-STAT, Inc.	Malden, MA, USA		
Laura	Spurchise		MPH	DM-STAT, Inc.	Malden, MA, USA		
Derek	Petersen		BS	DM-STAT, Inc.	Malden, MA, USA		
Gregory	Toland		MS	DM-STAT, Inc.	Malden, MA, USA		
Michael	Carmen		MS	DM-STAT, Inc.	Malden, MA, USA		
Cheri	Raffo		MPH	DM-STAT, Inc.	Malden, MA, USA		
Cindy	Mai		BA	DM-STAT, Inc.	Malden, MA, USA		
Jamie	Collins		PhD	DM-STAT, Inc.	Malden, MA, USA		
Patti	Folan			DM-STAT, Inc.	Malden, MA, USA		
Ingrid A.	Holm		MD	Boston Children’s Hospital	Boston, MA, USA		
David S.	Paterson		PhD	Boston Children’s Hospital	Boston, MA, USA		
Richard A.	Belliveau		BA	Boston Children’s Hospital	Boston, MA, USA		
Richard D.	Goldstein		MD	Boston Children’s Hospital	Boston, MA, USA		
Kevin G.	Broadbelt		PhD	Boston Children’s Hospital	Boston, MA, USA		
Kyriacos	Markianos		PhD	Boston Children’s Hospital	Boston, MA, USA		
Hanno	Steen		PhD	Boston Children’s Hospital	Boston, MA, USA		
Hoa	Tran		PhD	Boston Children’s Hospital	Boston, MA, USA		
Kristin	Rivera		MS	Boston Children’s Hospital	Boston, MA, USA		
Megan	Minter		MS	Boston Children’s Hospital	Boston, MA, USA		
Claire F.	Maggiotto		BS	Boston Children’s Hospital	Boston, MA, USA		
Kathryn	Schissler		DO	Boston Children’s Hospital	Boston, MA, USA		
Whitney	Adler		BA	Sanford Research	Sioux Falls, SD, USA		
Elizabeth	Berg		RN	Sanford Research	Sioux Falls, SD, USA		
Christa	Friedrich		MS	Sanford Research	Sioux Falls, SD, USA		
Jessica	Gromer		RN	Sanford Research	Sioux Falls, SD, USA		
Margaret	Jackson		BA	Sanford Research	Sioux Falls, SD, USA		
Luke	Mack		MA	Sanford Research	Sioux Falls, SD, USA		

Supplemental Online Content: Nonauthor Collaborators

*Indicates required information. Only first name, last name, and suffix will appear in PubMed.

*First Name and Middle Initial(s)	*Last Name	*Suffix (eg, Jr, III)	Academic Degrees	Institution	Location (city, state/province, country)	Role or Contribution, eg, chair, principal investigator	Group (if more than 1 Group listed in the byline) and/or Subgroup (eg, Steering Committee)
Bethany	Norton		MA	Sanford Research	Sioux Falls, SD, USA		
Liz	Swenson		RN	Sanford Research	Sioux Falls, SD, USA		
Deborah	Tobacco		MA	Sanford Research	Sioux Falls, SD, USA		
Amy	Willman		BS, RN	Sanford Research	Sioux Falls, SD, USA		
Deana A.	Him		PhD	Sanford Research	Sioux Falls, SD, USA		
Lisa B.	Robe			Sanford Research	Sioux Falls, SD, USA		
Mary	Berdahl		RN, BA	Sanford Research	Sioux Falls, SD, USA		
Donna	Black		AA	Sanford Research	Sioux Falls, SD, USA		
Jocelyn	Bratton		BS	Sanford Research	Sioux Falls, SD, USA		
Chaleen	Brewer		BS	Sanford Research	Sioux Falls, SD, USA		
Melissa	Berry		RD	Sanford Research	Sioux Falls, SD, USA		
Cathy	Christophersen		MA	Sanford Research	Sioux Falls, SD, USA		
Sue	Cote		LPN	Sanford Research	Sioux Falls, SD, USA		
Kari	Daron			Sanford Research	Sioux Falls, SD, USA		
Alexandra	Draisey		BSN, RN	Sanford Research	Sioux Falls, SD, USA		
Sara	Fiedler		MS	Sanford Research	Sioux Falls, SD, USA		
Kathy	Harris		RN, BSN	Sanford Research	Sioux Falls, SD, USA		
Lyn	Haug		RN, BSN, RNC-EFM	Sanford Research	Sioux Falls, SD, USA		
Lynn	Heath		BS, RDMS	Sanford Research	Sioux Falls, SD, USA		
Ann	Henkin		MS	Sanford Research	Sioux Falls, SD, USA		
Tara	Herman		RDMS	Sanford Research	Sioux Falls, SD, USA		
Jessica	Holsworth		BS	Sanford Research	Sioux Falls, SD, USA		
Kimberly	Lucia		MS	Sanford Research	Sioux Falls, SD, USA		
Laura	Medler		RN	Sanford Research	Sioux Falls, SD, USA		
Libby	Nail		MD	Sanford Research	Sioux Falls, SD, USA		
Amber	Ogaard		BS	Sanford Research	Sioux Falls, SD, USA		
Debby	Olson		JD	Sanford Research	Sioux Falls, SD, USA		
Mary	Reiner		BA, CCRP	Sanford Research	Sioux Falls, SD, USA		
Carol	Robinson		RN	Sanford Research	Sioux Falls, SD, USA		

Supplemental Online Content: Nonauthor Collaborators

*Indicates required information. Only first name, last name, and suffix will appear in PubMed.

***First Name and Middle Initial(s)**	***Last Name**	***Suffix (eg, Jr, III)**	Academic Degrees	Institution	Location (city, state/province, country)	Role or Contribution, eg, chair, principal investigator	Group (if more than 1 Group listed in the byline) and/or Subgroup (eg, Steering Committee)
Brooke	Schmitt		BSN, RN	Sanford Research	Sioux Falls, SD, USA		
Monique S.	Bob		RN	Sanford Research	Sioux Falls, SD, USA		
Lacey	Stawarski		MD	Sanford Research	Sioux Falls, SD, USA		
Sherri T.	Fingers		RN	Sanford Research	Sioux Falls, SD, USA		
Rachel	Thies		MD	Sanford Research	Sioux Falls, SD, USA		
Mary	Thum		MS	Sanford Research	Sioux Falls, SD, USA		
Elizabeth	Wheeler		MPH	Sanford Research	Sioux Falls, SD, USA		
Lisa W.	Bull		MS	Sanford Research	Sioux Falls, SD, USA		
Steve W.	Hat		AAN	Sanford Research	Sioux Falls, SD, USA		
Amy	Wilson		RN	Sanford Research	Sioux Falls, SD, USA		
Neva	Zephier		MPH	Sanford Research	Sioux Falls, SD, USA		
Misti	Zubke		BS, RDMS	Sanford Research	Sioux Falls, SD, USA		
Heidi	Bittner		MD	Sanford Research	Sioux Falls, SD, USA		
Jeffrey	Boyle		MD	Sanford Research	Sioux Falls, SD, USA		
Donna	Gaspar		RN, MA	Sanford Research	Sioux Falls, SD, USA		
Cheryl	Hefta		MS, RN	Sanford Research	Sioux Falls, SD, USA		
Michael	McNamara		MD	Sanford Research	Sioux Falls, SD, USA		
Karna	Colby		MD	Sanford Research	Sioux Falls, SD, USA		
Kent	Donelan		MD	Sanford Research	Sioux Falls, SD, USA		
Don	Habbe		MD	Sanford Research	Sioux Falls, SD, USA		
Catherine	Stoos		MD	Sanford Research	Sioux Falls, SD, USA		
H. Eugene	Hoyme		MD	Sanford Research	Sioux Falls, SD, USA		
Amy	Mroch		MS	Sanford Research	Sioux Falls, SD, USA		
Erna	Carstens		RN	Stellenbosch University	Stellenbosch, South Africa		
Lucy	Brink		MS	Stellenbosch University	Stellenbosch, South Africa		
Lut	Geerts		MD, MRCOG	Stellenbosch University	Stellenbosch, South Africa		

*Indicates required information. Only first name, last name, and suffix will appear in PubMed.

*First Name and Middle Initial(s)	*Last Name	*Suffix (eg, Jr, III)	Academic Degrees	Institution	Location (city, state/province, country)	Role or Contribution, eg, chair, principal investigator	Group (if more than 1 Group listed in the byline) and/or Subgroup (eg, Steering Committee)
Greetje	de Jong		MBChB, MMed, MD	Stellenbosch University	Stellenbosch, South Africa		
Pawel	Schubert		FCFor Path, MMed	Stellenbosch University	Stellenbosch, South Africa		
Shabbir	Wadee		MMed	Stellenbosch University	Stellenbosch, South Africa		
Johan	Dempers		FCFor Path	Stellenbosch University	Stellenbosch, South Africa		
Elsie	Burger		FCFor Path, MMed	Stellenbosch University	Stellenbosch, South Africa		
Janetta	Harbron		PhD	Stellenbosch University	Stellenbosch, South Africa		
J. David	Nugent		MA	Columbia University Irving Medical Center	New York, NY, USA		
Carmen	Condon		BA	Columbia University Irving Medical Center	New York, NY, USA		
Joseph	Isler		PhD	Columbia University Irving Medical Center	New York, NY, USA		
Margaret C.	Shair		BA	Columbia University Irving Medical Center	New York, NY, USA		
Yvonne	Sininger		PhD	Columbia University Irving Medical Center	New York, NY, USA		
Chuan-Ming	Li		MD, PhD	NIH	Bethesda, MD, USA		
Caroline	Signore		MD, MPH	NIH	Bethesda, MD, USA		
Ken	Warren		PhD	NIH	Bethesda, MD, USA		
Elizabeth	Thom		PhD	George Washington University	Washington, DC, USA		
Phillip	Cato		PhD	Duke University	Durham, NC, USA		
James W.	Collins		MD, MPH	Northwestern University	Evanston, IL, USA		

Supplemental Online Content: Nonauthor Collaborators

*Indicates required information. Only first name, last name, and suffix will appear in PubMed.

***First Name and Middle Initial(s)**	***Last Name**	***Suffix (eg, Jr, III)**	Academic Degrees	Institution	Location (city, state/province, country)	Role or Contribution, eg, chair, principal investigator	Group (if more than 1 Group listed in the byline) and/or Subgroup (eg, Steering Committee)
Terry	Dwyer		MD, MPH	University of Oxford	Oxford, United Kingdom		
George	Macones		MD	Washington University	St. Louis, MO, USA		
Philip A.	May		PhD	University of North Carolina at Chapel Hill	Chapel Hill, NC, USA		
Richard M.	Pauli		MD, PhD	University of Wisconsin	Madison, WI, USA		
Raymond W.	Redline		MD	University Hospitals Case Medical Center	Cleveland, OH, USA		
Michael	Varner		MD	University of Utah Health Sciences Center	Salt Lake City, UT, USA		
Dale	Hereld		PhD	National Institute on Alcohol Abuse and Alcoholism (NIAAA)	Rockville, MD, USA		
